# Capillary Electrophoresis Mass Spectrometry Interfacing via Multifunctional Vibrating Sharp Edge Ionization Spray for the Simultaneous Delivery of an Auxiliary Flow, Analyte Mixing, and Fluid Nebulization

**DOI:** 10.1021/acs.analchem.5c04794

**Published:** 2026-02-27

**Authors:** Yousef S. Elshamy, Lisa A. Holland, Eric L. Corley

**Affiliations:** C. Eugene Bennett Department of Chemistry, 5631 West Virginia University, Morgantown, West Virginia 26505, United States

## Abstract

A new method of connecting capillary electrophoresis (CE) to mass spectrometry (MS) is introduced in which a vibrating sharp edge spray ionization (VSSI) probe is adapted to deliver a secondary fluid directly at the capillary electrophoresis surface. In VSSI, acoustic streaming at a sharp edge converts solutions into an aerosol. Consequently, a superimposed electric field is not required to nebulize fluid as is the case for electrospray ionization. In this report, a directed VSSI auxiliary flow assists in analyte transfer to the MS, making it amenable to electrophoretic separations that have a low electroosmotic bulk flow. Unlike a coaxial sheath, a directed auxiliary flow can be used with nonconductive liquids because the superimposed fluid is not integral to the process of electrophoresis grounding. An order of magnitude improvement in analyte signal is realized when deionized water is used for the supplementary liquid. In addition to the multifunctional role of VSSI for delivery of fluid, mixing with analyte, and nebulization, a self-aligning grounding cap is described for use with standard fused silica separation capillaries that have a blunt cut at the end. These features enable coupling to a commercial CE instrument. The direct CE-VSSI-MS interface is compatible with background electrolytes maintained at acidic or neutral pH and even composed of 200 mM ammonium acetate. Separations are demonstrated with cationic beta-blockers, amino acids, peptide standards, a peptide mixture from a chymotrypsin digestion of transferrin, and a 17-residue peptide monomer and homodimer derived from Huntington’s protein that is implicated in protein aggregation.

## Graphical abstract

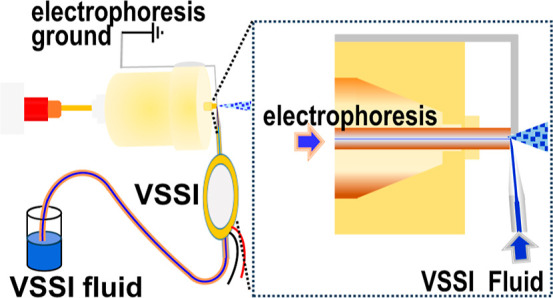

Capillary electrophoresis is an efficient microscale separation technique ideal for fast analyses of small volume samples. When combined with mass spectrometry, this method can identify molecules based on both migration time and molecular mass. As a result, this analytical technique is pivotal for analyses that benefit from good separation efficiency and are also challenged by a limited availability of sample or small sample volume constraints. The current method of coupling capillary electrophoresis in-line to mass spectrometry is through electrospray ionization, which like electrophoresis, is a voltage driven process. Different approaches have been devised to decouple the separation and ionization currents through metal leads that contact the spray directly or through conductive liquids that penetrate porous fractures near the spray.^1^ An additional consideration is that the inherently low fluid flow rates (i.e., subnanoliter to submicroliter per minute) of capillary electrophoresis can be incompatible with the requirements for the electrospray fluid consumption. Moreover, the capillary electrophoresis flow rate is dependent upon many factors that affect the double layer on the capillary surface including the applied voltage, pH, ionic strength and analyte adsorption on the silica wall. Unlike liquid chromatography, in capillary electrophoresis the rate at which fluid that carries the analyte is delivered to the end of the separation capillary varies according to the separation parameters that are selected by the user. As a result, the conditions most appropriate for the separation may not be well suited to sustain the electrospray process. This issue can be resolved in electrospray interfacing by using a pressure assisted-flow in the separation, although this approach can reduce the separation efficiency.^2^

A number of sensitive electrospray ionization sources are reported in the literature that accommodate the liquid flow rate of capillary electrophoresis with sheathless interfacing. Examples include the tapered-tip^3^ and the commercialized porous etched tip with a conductive liquid to introduce the electrical connection to the separation and the electrospray.^4^ Alternatively, a wide variety of interfaces incorporate sheath fluid, which flows around the capillary at subnanoliter to submicroliter flow rates to augment the capillary electrophoresis flow. Examples include separation capillaries inserted into tapered tips with sheath flows delivered through 3D printed channels^5^ and through the commercialized cross connection which supports electrokinetic pumping of the sheath flow liquid.^6^

Vibrating sharp-edge spray ionization (VSSI) is a recent method that differs from electrospray ionization.^7^ The VSSI operates in the absence of the electric field that is typically used to drive electrospray processes. In VSSI, acoustic streaming at vibrating sharp edges leads to focused and intense fluid motions which transform liquid streams to an aerosol. The fluid motions at the VSSI edge produce vortices at a 90° angle to the edge.^8^ A VSSI probe with a pulled tip can be used to deliver a liquid. It can also be used to nebulize liquid in contact with the probe tip. In this report a VSSI probe is created that simultaneously delivers an auxiliary flow to the electrophoresis, mixes this supplemental fluid with the liquid expelled from the electrophoresis capillary, and aerosolizes the fluid mixture so that it can be introduced into a mass spectrometer. The elimination of an electric field to drive nebulization and the introduction of direct mixing between the separated analytes and a supplementary flow provide new strategies to address two challenges to capillary electrophoresis-mass spectrometry interfacing.

For the first time, a capillary electrophoresis interface is reported with the auxiliary flow delivered through a spray source which is placed in contact with the analyte exiting the separation capillary. An important feature of the interface is the electrophoresis ground, which is incorporated through a universal capillary holder, terminating the electrophoresis circuit at the capillary outlet. These two design elements eliminate the need for a conductive solvent to complete the electrophoresis circuit. With direct VSSI, grounding is simplified with an end-cap that can be placed on any standard fused silica capillary that has been trimmed to create a flat surface. Although VSSI has been applied to capillary electrophoresis in a sheathless,^9^ nanoflow sheath^10^ and microflow sheath^11^ format, the approach outlined in this report is simpler to integrate into a commercial capillary electrophoresis instrument. The new design is significant and innovative because a nonconductive (i.e., deionized water) auxiliary fluid can be used to modulate the delivery of analyte to the MS inlet, and a slip on fitting can be removed and replaced to create the electrophoretic ground for any blunt cut fused silica separation capillary.

This report demonstrates the analyte transport from the separation capillary to the nebulized drops through fluid mixing. The details of the direct VSSI interface, including the integration on a commercial capillary electrophoresis instrument are outlined. The performance of the direct application of a supplemental liquid is assessed using a fluid that is identical to the background electrolyte, and found to be equivalent to the previously reported sheathless VSSI and nanoflow sheath VSSI which were both coupled to a laboratory-built capillary electrophoresis instrument. For the direct VSSI auxiliary flow, the ionization is improved when deionized water is used as the supplemental liquid. The utility of the interface is demonstrated using a commercial instrument with cationic beta-blockers as well as a variety of peptide mixtures. The signal obtained with direct flow VSSI does not differ for peptide separations obtained in background electrolyte that is acidic or maintained at a neutral pH. This enables the analyses of peptides under more native conditions with a neutral, higher ionic strength background electrolyte with an auxiliary flow composed of deionized water and is applied to a peptide fragment from Huntington’s protein that is reported to form homodimers under physiological conditions.

## Materials and Methods

### VSSI

The VSSI probe is fabricated using hollow glass precision capillary tubes (0.4 mm i.d., 7.5 cm Drummond Scientific Co, Broomall, PA). The capillary tubes are pulled using a laser puller (Sutter Instrument Company, Novato, CA) with the following parameters: line 1: HEAT = 475, FIL = 4, VEL = 60, DEL = 130, PUL = 80, and line 2: HEAT = 650, FIL = 4, VEL = 60, DEL = 130, PUL = 40. The pulled glass tubes are trimmed to a tip diameter between 40 and 75 μm and attached to the underside of a piezoelectric transducer (7BB-27-4 L0, diameter = 27 mm, Murata, Duluth, GA) using 5 min epoxy and allowed to dry overnight. The epoxy is applied to the end of a 40 cm long, 30 μm i.d., 360 o.d. fused silica capillary (TSP030375, Polymicro Technologies, Phoenix, AZ), leaving the last 2 mm of the capillary uncoated to avoid clogging. This end of the capillary is inserted into the pulled glass probe up to the taper within the probe. The end of the glass probe is sealed externally with epoxy to prevent backflow of the auxiliary fluid, and the assembly is dried overnight. The piezoelectric transducer is connected to a frequency generator (DDS signal generator/counter Koolertron, Hong Kong Karstone Technology Co, Hong Kong) and an operational amplifier (OPA541, Taidacent, Shenzhen Taida Century Technology Co., Ltd., Shenzhen China). A square wave is applied with frequency and amplitude ranging from 90 to 97 kHz and 10 to 12 V_pp_, respectively. For each device, the frequency and amplitude are adjusted within this range to achieve a visually intense microdroplet plume.

### Capillary Electrophoresis

The grounding electrode is formed from a size 0 stainless steel entomology insect pin bent at two 90° angles and fixed to a polymer fitting (CapTite Bonded-Port Connector #360–400, LabSmith, Livermore, CA) using epoxy. The electrode is fixed approximately 0.5 cm above the hole of the polymer fitting. The separation capillary, cut with a flat surface, is inserted in a CapTite fitting (#C360, LabSmith) and this ferrule is then tightened into the CapTite port connector, which positions the orifice of the separation capillary near the grounding electrode. A 25 μm i.d. 360 μm o.d. capillary (TSP025375, Polymicro Technologies) 27 or 85 cm is used for the lab-built or commercial electrophoresis instrument, respectively. For the separations performed at neutral pH, prior to use, the capillary is flushed at 345 kPa (50 p.s.i.) with 0.1 N ammonium hydroxide, deionized water, and background electrolyte for 30, 15, and 30 min, respectively. For the separations performed at acidic pH, prior to use, the capillary is flushed at 345 kPa (50 p.s.i.) with 2% formic acid for 30 min. The capillary is flushed in between each electrophoresis separation up to 3 min with background electrolyte. The cartridge temperature is 25 °C. The P/ACE MDQ capillary electrophoresis instrument (formerly Beckman Coulter, Sciex, Marlborough, MA) is controlled by 32 Karat and fitted with an external detector adapter (A61216, Sciex) for MS detection using the firmware settings prescribed by the manufacturer.

## Results and Discussion

In VSSI rapid fluid movement (m/s velocity) is induced around vibrating sharp edges as acoustic energy dissipates into the surrounding liquid.^12^ When the fluid contacts the vibrating edges, this intense fluid motion transforms the bulk liquid into smaller drops.^7,13^ As depicted in Figure 1A, the VSSI design in this report directs a supplemental flow at the electrophoresis outlet to carry the analyte to the MS inlet. This additional fluid is particularly important when the electroosmotic flow rate of the capillary electrophoresis is at or below 70 nL/min.^9,10^ The VSSI probe, which delivers 650 ± 10 nL/min (*n* = 3 measurements at *P* = 138 kPa), is positioned at the orifice to direct and mix the auxiliary flow with fluid migrating from the electrophoresis capillary. The mixed solution is converted to aerosol. This direct interface is a significant advance over prior capillary electrophoresis VSSI interfacing because it enables the user to integrate blunt cut, standard separation capillaries and it decouples the electrophoresis ground from a superimposed flow.

**Figure 1:**
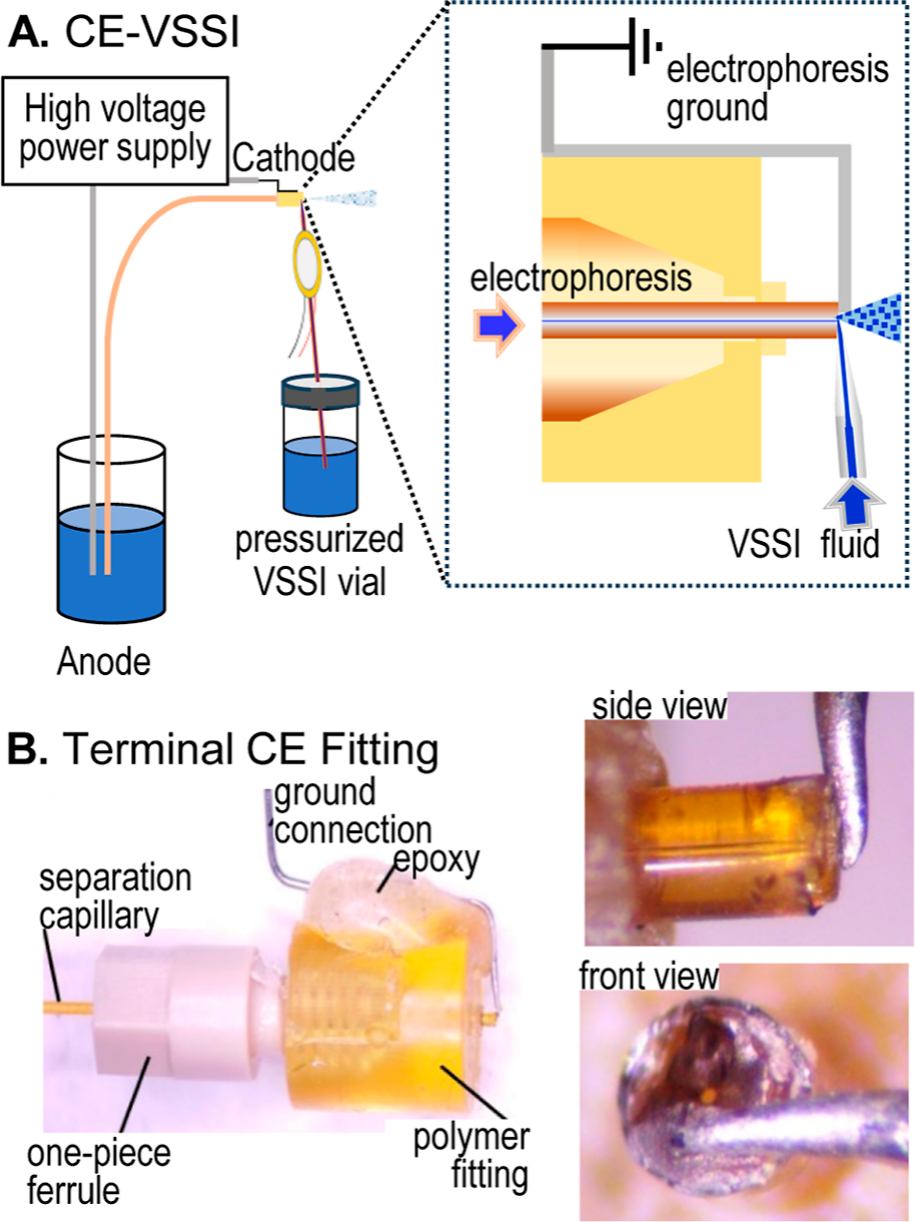
(A) Conceptual depiction of the direct VSSI interface to capillary electrophoresis. (B) Images of the cap used to ground the electrophoresis including a magnified side and front view of the electrode in contact with the separation capillary.

After the analytes are sorted by the electrophoresis step, the separated analytes are mixed with the auxiliary fluid, nebulized by the VSSI probe, and transferred to the mass spectrometry inlet. The fluid mixing is visualized through fluorescence measurements of the drops generated when Cy5 and fluorescein are pushed through the separation capillary and VSSI probe, respectively. The VSSI drops formed after mixing at the interface are captured in mineral oil, imaged using a fluorescence microscope with appropriate filters, and converted to grayscale, revealing a similar dye ratio in the drops (Figures S1, S2A–C and summarized in Table S1 in the Supporting Information).

### CE-VSSI-MS Interface

In this novel VSSI design, the separation capillary is grounded with a fitting that positions the capillary orifice near a grounding electrode in a prealigned position (Figure 1B) and brought out of the instrument using an external adapter (Figure 2A). This polymer fitting and a ferrule are slipped over the capillary and tightened. The grounding electrode is constructed from stainless steel rather than platinum, making it cost-effective and less susceptible to breaking. A Nanospray Flex interface (ES071, Thermo Fisher Scientific) is used to mount the separation capillary and to position it 2 mm from the MS inlet. An additional micromanipulator (MT-XYZ, Newport Corp., Irvine, CA) is added (Figure 2A) to position the VSSI probe near the orifice of the separation capillary (Figure 2B). After visually placing the VSSI probe at the end of the separation capillary using a magnifying camera (Figure 2B), the probe position is nominally adjusted to achieve the highest signal possible with direct infusion of propranolol.

**Figure 2:**
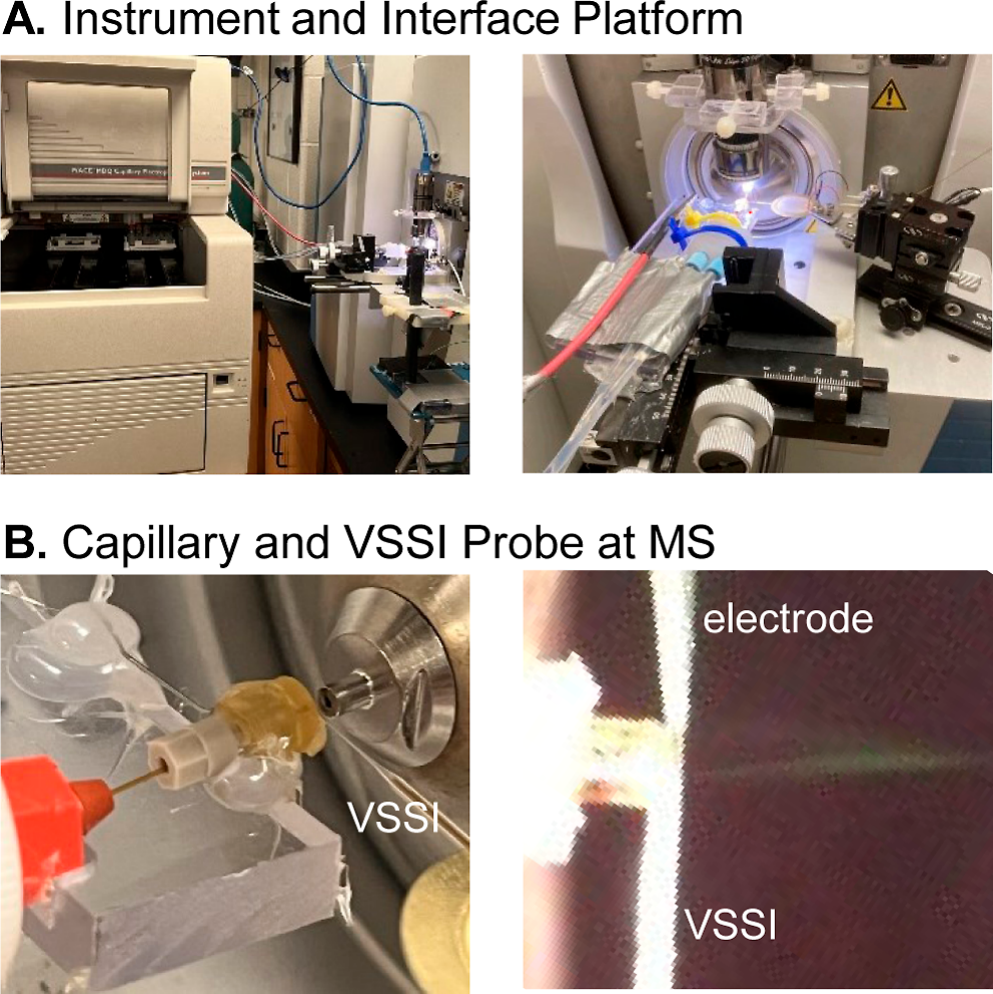
(A) Images of the commercial capillary electrophoresis instrument and the platform holding the vibrating sharp-edge spray ionization (VSSI) probe and separation capillary. (B) Magnified photos of the separation capillary, VSSI probe and grounding electrode for electrophoresis.

### Comparison of Direct VSSI to Nanoflow Sheath VSSI

In order to compare the performance of the direct VSSI to the previously published nanoflow sheath VSSI,^10^ the detection limit is evaluated with the same lab-built instrumentation, including the electronic timer for sample injection, power supply, and capillary length. A 10 nM sample of pindolol dissolved in 50 mM ammonium acetate is electrokinetically injected (20 kV, 2 s) and separated using a 27 cm long, 25 μm inner diameter separation capillary. The limit of detection, calculated as described previously,^9^ for the direct VSSI is 1.8 ± 0.8 nM (*n* = 3, see Figure S3A in the Supporting Information). This is not significantly different (student’s *t*-test, ρ = 0.05, data normality confirmed) from the detection limit of 6 ± 3 nM calculated for the nanoflow sheath design^10^ (see Figure S3B in the Supporting Information). Both the previously reported sheath flow^10^ and the direct VSSI auxiliary flow used the same background electrolyte. The signal intensity of the direct flow VSSI configuration is improved by an order of magnitude when injection stacking is employed by maintaining the same injection conditions, but diluting the analyte in a lower ionic strength ammonium acetate, causing the peak area to increase from 30,000 ± 10,000 for a 10 nM pindolol solution in 50 mM ammonium acetate to 110,000 ± 9000 for a 1 nM pindolol solution in 1 mM ammonium acetate. An additional enhancement is achieved by changing the composition of the fluid pumped through the VSSI probe from the background electrolyte to deionized water, which increases the peak area even further to 400,000 ± 100,000 for the same 1 nM pindolol sample. Low ionic strength aqueous solutions have reduced ion suppression when used previously in a VSSI sheath flow system.^14^ The use of a deionized water sheath may enhance the signal of the direct VSSI design by reducing ions from the electrophoresis background electrolytes that compete with the analyte during ionization.

### Integration and Evaluation in a Commercial Instrument

As shown in Figure 2, the direct flow VSSI can be used in a commercial instrument equipped with an external detector adapter and an 85 cm, 25 μm inner diameter capillary. The system is evaluated with solutions of pindolol dissolved in the background electrolyte at a concentration of 20, 100, and 200 nM, injected using pressure, separated, and detected using a matched auxiliary flow. The resulting calibration curve of *Y* = (1.06_5_ ± 0.01_7_ × 10^2^)*X* + (1._5_ ± 2._3_ × 10^2^), is fit effectively with linear regression (*R* ^2^ = 0.9997). The limit of detection estimated with this curve is 6 nM. This detection limit is equivalent to 8 attomoles (i.e., 2 femtograms) of pindolol based on a calculated injection volume of 1.3 nanoliters, given the 4 s 28 kPa (4.0 psi) sample injection and an estimated flow velocity of 0.064 cm/s at 28 kPa derived from a flow velocity of 1.2 cm/s measured at a 520 kPa (75 psi). The effect of the composition of the auxiliary fluid is also observed in the commercial instrument with the direct infusion of 400 nM pindolol dissolved in 50 mM ammonium acetate. As the composition of the auxiliary fluid is changed from 50 mM to 2.5 mM to no ammonium acetate the signal increases from 1.2 × 10^4^ to 3.1 × 10^4^ to 3.7 × 10^4^, respectively.

A separation of β-blockers in the presence of a forward electroosmotic flow is shown in Figure 3. The precision in migration time and peak area is 0.5 and 20% relative standard deviation, respectively (see Table S2 in the Supporting Information). This migration time precision is comparable to results obtained using a P/ACE MDQ instrument with optical detection,^10^ and is improved (i.e., 8 times lower) when compared to a lab-built instrument with a VSSI nanoflow sheath^10^ as a result of automation and temperature control of the commercial instrument. With the commercial instrument coupled to direct VSSI, the precision in peak area improves further when peak overlap is mitigated. Replicate separations (*n* = 5) of sample containing only pindolol at a concentration of 200 nM, with a pressure-based injection and no sample stacking, have a precision in migration time (6.94 ± 0.04 min) and peak area (67,000 ± 8000), of 0.6 and 10%, respectively.

**Figure 3:**
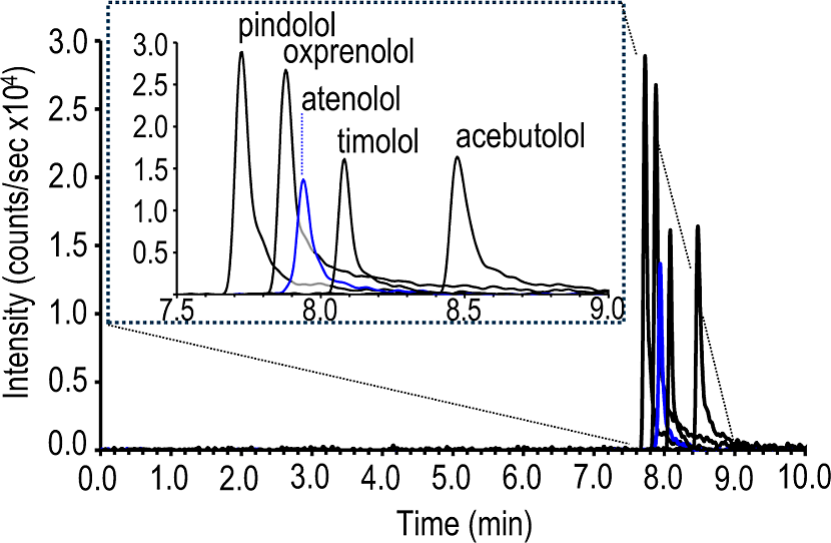
Electropherogram of 10 nM β-blockers achieved with an 85 cm, 25 μm inner diameter capillary, with 50 mM ammonium acetate (pH 6.8) at an applied voltage of 25 kV, and current of 5.9 μA. The sample, diluted in 1 mM ammonium acetate, is injected (10 kV, 12 s) with stacking. The auxiliary flow (650 nL/min) is deionized water.

The applicability of this design with a suppressed electroosmotic flow is demonstrated with separations of amino acids (Figure 4A, and Table S3 in the Supporting Information) and peptides (Figure 4B and Table S4 in the Supporting Information) achieved with a background electrolyte composed of 2% formic acid at a pH of 2. Under these conditions the analyte is transferred to the VSSI by electrophoretic migration directly into the supplemental flow composed of deionized water. When the amino acid separation is compared to a previously reported nanoflow sheath system,^10^ the peak areas are approximately 2 orders of magnitude higher, although there are differences in the electrophoresis systems and separation. The capillary electrophoresis instrument in the prior report^10^ was constructed in the lab, leading to the use of a 30 cm, 25 μm i.d. capillary operated in the absence of thermal control with an applied voltage of 12 kV (8.2 μA), an electrokinetic sample injection of 3 s, 20 kV for amino acids dissolved in 0.004% formic acid, a background electrolyte of 2% formic acid, and a 900 nL/min sheath flow composed of 2% formic acid. In current system, the deionized water auxiliary flow enhanced the signal by reducing competitive ionization. When a sheath of 2% formic acid is used with a 2% formic acid background electrolyte, the peak area observed for arginine is 20 times lower.

**Figure 4:**
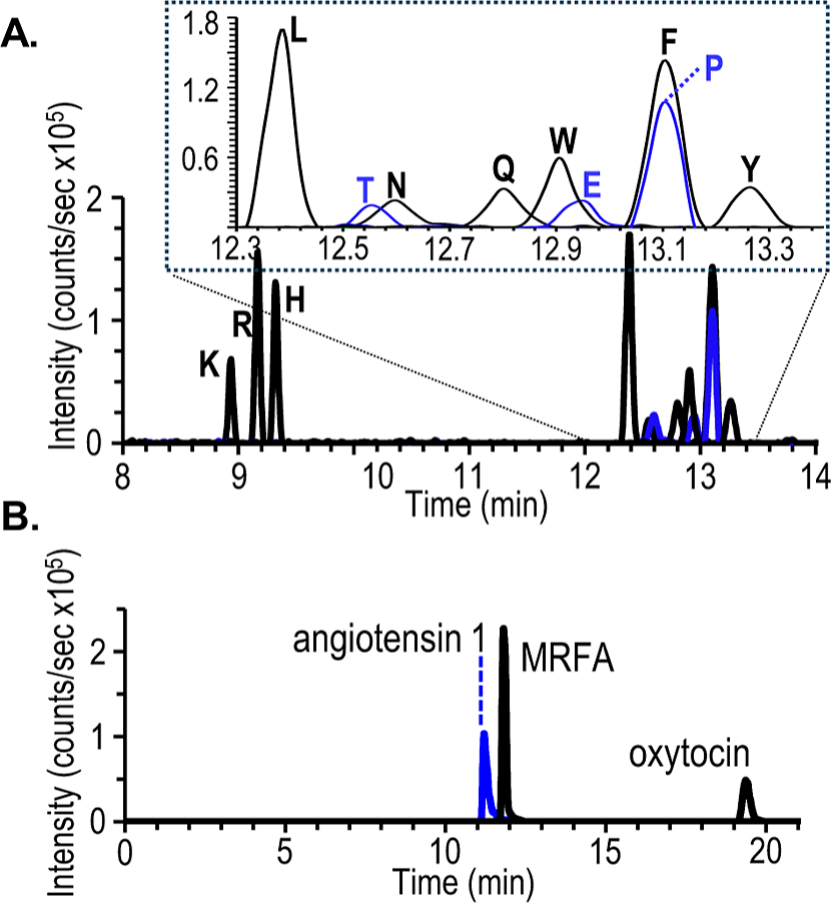
Electropherogram obtained with a suppressed electroosmotic flow using a background electrolyte of 2% formic acid at pH 2. The amino acids in trace (A) are 0.5 μM (K, R, H) or 2.5 μM (L, N, T, Q, W, E, F, P, Y) diluted in 0.004% formic acid injected (10 kV, 6 s) with stacking. Peptides in traces (B) are 50 μM MRFA, angiotensin I, and oxytocin diluted in water and inject at 5 psi, 4 s. Other separation conditions are as described in Figure 3.

To evaluate the effect of pH on the MS detection, the peptides separated with a suppressed electroosmotic flow (Figure 4B) are compared to those obtained with an active electroosmotic flow achieved using ammonium acetate at pH 6.78 (see Figure S4 and Table S4 in the Supporting Information). Separations in both acidic and near-neutral background electrolytes are dissolved in deionized water, and injected with pressure (5 psi, 4 s). The combined peak areas for the +1 and +2 charge states for each peptide across 4 electrophoresis separations obtained under acidic or near neutral conditions are not statistically different (student’s *t*-test, ρ = 0.05). It should be noted that sodium adducts are observed with each peptide at both pH values and the combined peak areas do not differ when the adducted masses are also considered in the comparison. The direct VSSI interface is also applied to separations of peptides resulting from the chymotrypsin digestion of transferrin using acidic as well as near neutral background electrolyte. The use of a low specificity chymotrypsin, which cleaves at five residues (F, Y, W, M, L) on transferrin that has been reduced and alkylated with iodoacetamide is predicted by Expasy PeptideMass to produce 136 fragments.^15^ Analysis of the traces, summarized in Figure S5A,B and Table S5 in the Supporting Information, identify that only 85 of these peptides are within a scanned mass range of 300–3000 Da and of these 68 and 54 of the predicted masses are detected with the acidic and neutral background electrolytes, respectively. These results further indicate that the background electrolyte pH can be selected based on the goals of the electrophoresis application rather than the MS detection.

The capillary electrophoresis-VSSI system is also compatible with neutral background electrolyte at higher ionic strength to separate peptides that interact under physiological conditions. For example, a 17 amino acid peptide on the N-terminus of Huntington’s protein (Nt^17^) is reported to self-aggregate more effectively in the presence of polyproline. A mixture of the Nt^17^ and a 10 residue polyproline (polyP_10_) peptide are separated in a background electrolyte composed of 200 mM ammonium acetate and 20 mM methyl morpholine buffered to pH 7 (see Figure 5). With the MS detection, the dimer of Nt^17^ is observed in the presence of the Nt^17^ and polyP10 monomers. These results demonstrate that the capillary electrophoresis-VSSI-mass spectrometry system can be applied to systems that require near neutral and even high ionic strength solutions to recapitulate peptide interactions observed under physiological conditions.

**Figure 5:**
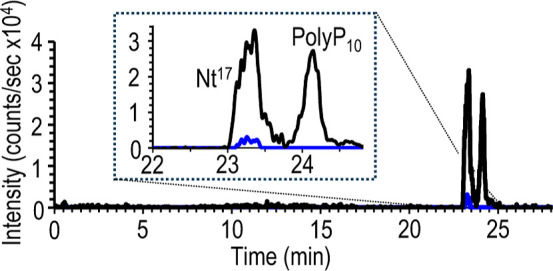
Electropherogram of monomeric polyP_10_ and Nt^17^ peptides (black trace) and dimeric Nt^17^ (blue trace). The peptides are separated with a background electrolyte of 200 mM ammonium acetate and 20 mM methyl morpholine adjusted to pH 7 and the auxiliary solution is deionized water.

## Conclusions and Future Directions

The VSSI interface in this report provides the same analytical performance as previous sheath flow and sheathless capillary electrophoresis VSSI systems. With the direct auxiliary flow VSSI a conductive or nonconductive fluid can be used to maintain the fluid flow rate to the MS at 650 nL/min. The applications in this report demonstrate that the VSSI is compatible with suppressed or active electroosmotic flow and can be operated at acidic, neutral and high ionic strength background electrolytes in the electrophoresis.

The direct interface enables straightforward coupling of VSSI to any blunt cut fused silica capillary with a 360 μm outer diameter. The design is simple to install and can be adapted to any commercial electrophoresis instruments that allow for external detection. The VSSI probe described in this report was used repeatedly without being clogged, although highly purified deionized water and mass spectrometry grade reagents were used to formulate the auxiliary fluid. The polymer sleeve with the electrophoresis ground and the VSSI probe are used repeatedly for analyses, but can be considered disposable based on the low cost. A consideration of the current design is that the probe is manually aligned with the capillary orifice and the grounding electrode. If the probe is aligned improperly, the signal intensity will vary, and a lower precision will be observed for the area. Future studies with the direct VSSI interface will involve expanded applications to native mass spectrometry beyond the Nt^17^ system, improved methods to prealign the VSSI probe and capillary, and a direct comparison of the VSSI direct interface with commercially available electrospray.

## Supplementary Material



## Notes

Data obtained with CE-VSSI-MS, summaries of ionization intensities (PDF)
