# Supplementary material for: Capillary Electrophoresis Mass Spectrometry Interfacing via Multifunctional Vibrating Sharp Edge Ionization Spray for the Simultaneous Delivery of an Auxiliary Flow, Analyte Mixing, and Fluid Nebulization
Source: Anal Chem. 2026 Feb 27;98(9):6462–7. doi: 10.1021/acs.analchem.5c04794 (PMC12980493; doi:10.1021/acs.analchem.5c04794)
Supplement: Supplementary file 1 [file ac5c04794_si_001.pdf]

SUPPORTING INFORMATION

Capillary Electrophoresis Mass Spectrometry Interfacing Via Multifunctional Vibrating Sharp Edge Ionization Spray for the Simultaneous Delivery of an Auxiliary Flow, Analyte Mixing, and Fluid Nebulization

Yousef S. Elshamy and Lisa A. Holland,[1*] Eric L. Corley

[1]C. Eugene Bennett Department of Chemistry, West Virginia University, Morgantown, WV 26505

*Corresponding author contact information: Lisa.Holland@mail.wvu.edu

ABSTRACT This material includes data that demonstrates the figures of merit obtained with capillary electrophoresis (CE) separations and UV absorbance or mass spectrometry (MS). Raw data that is not in this document can be found in the following data archive: Holland, Lisa (2026), "Data for: Capillary Electrophoresis Mass Spectrometry Interfacing Via Multifunctional Vibrating Sharp Edge Ionization Spray for the Simultaneous Delivery of an Auxiliary Flow, Analyte Mixing, and Fluid Nebulization", Mendeley Data, V1, doi: 10.17632/52t496phd3.1

TABLE OF CONTENTS

Page S-1, Title of the article, authors names and affiliations, abstract and table of contents
Page S-2, Materials and Methods
Page S-3, Figure S1: Conceptual depiction of fluorescent measurement of mixing A
Page S-4, Figure S2A. Fluorescein imaged drops converted to grayscale
Page S-5, Figure S2 B. Cy5 imaged drops converted to grayscale
Page S-6, Table S1. Summary of the ratio of Cy5 to fluorescein grayscale intensity and Figure S2C denoting drop number associated with Table S1
Page S-7, Figure S3A Data Summary Direct VSSI LODs calculated using Signal and Noise
Page S-8, Figure S3B Data Summary of Previously reported Nanoflow Sheath original data used to calculate the LOD using Signal and Noise
Page S-9, Table S2. Migration times and peak areas of β-Blockers
Page S-10, Table S3. Migration and peak areas of amino acids
Page S-11, Table S4. Peak areas of peptide standards with acidic or neutral background electrolyte
Page S-12, Figure S4. Separation of peptide standards with a neutral background electrolyte
Page S-13, Figure S5. Separation of digested transferrin with an acidic or neutral pH background electrolyte
Page S-14, Table S5. Transferrin digest peptide mass data using acidic and near-neutral background electrolyte
Page S-15, Table S5 continued
Page S-16, Table S5 continued
Page S-17, Author credit statement

Materials and Methods

Chemicals and Reagents. Acebutolol (A-3669), amino acids (LAA-21), ammonium acetate (431311), angiotensin I acetate salt (A9650), atenolol (A-7655), α-chymotrypsin (C4129), dithiothreitol (3860-OP), fluorescein sodium salt (F-6377), iodoacetamide (I615), oxytocin acetate salt hydrate (O6379), pindolol (P-0778), propranolol (P-0884) and timolol (T-6394), tolmetin (T-6779), transferrin (T8158), mineral oil (M5904) are from Millipore Sigma (Burlington, MA). Nt17 (MATLEKLMKAFESLKSF) and polyP (Ac-P10 -NH 2) are from GenScript Biotech (Piscataway, New Jersy). Oxprenolol (156023) is from ICN Biomedicals Inc. (Aurora, OH). Formic acid (A13285) and 4-methylmorpholine (99%, AAA12158AP) are from Thermo Fisher Scientific (Waltham, MA). Ammonium hydroxide (BDH3016) and tris hydrochloride (4103-01) are from VWR Analytical (Radnor, PA). Disulfo Cy5 carboxylic acid 98% is obtained from APExBIO Tech LLC (Houston, TX). Deionized water (18 MΩ/cm) is obtained from an Elga Purelab and Veolia Chorus water system (Lowell, MA).

Transferrin Digestion. The transferrin is diluted using 100 mM Tris HCl at pH 8. The protein is desalted using an Amicon® ultra centrifugal filter 10 kDa molecular weight cutoff filter (UFC501008, Millipore Sigma) by exchanging the solution with approximately 1 mL of the pH 8 100 mM Tris HCl 2 times and then concentrating the protein to approximately 0.1 mL. In the final step, the 100 Tris HCl buffer is exchanged using 100 mM Tris HCL containing 6 M urea to denature the protein. For this report, the protein concentration after cleaning up and buffer exchanging to a volume of 70 μL was determined to be 37.69 μg/μL by using a NanoDrop™ 1000 spectrophotometer (Thermo Fisher Scientific). The reduction and alkylation is performed using reagents dissolved in 100 mM Tris HCl at pH 8. The sample is reduced at 37 °C for 2 hours using a dithiothreitol (DTT) solution at a molar ratio of 1:40 (protein: DTT). After reducing the disulfide bonds, the protein is alkylated using an iodoacetamide solution at a molar ratio of 1:80 (protein: iodoacetamide). This reaction proceeded in darkness for 2 h in ice. Next, a 1:40 molar excess of cysteine solution (protein:cysteine) is added and the reaction proceeds for 30 min at room temperature to quench the alkylation reaction. In order to reduce the urea, DTT, cysteine and other reagents that may adversely affect the MS analysis, the sample is exchanged 4 times using 100 mM 4-methylmorpholine also containing 10 mM calcium chloride and adjusted at pH 8 with acetic acid. The addition of calcium is necessary to activate the chymotrypsin. The protein concentration after the buffer exchange process is determined again using the Nanodrop Spectrophotometer. The chymotrypsin is prepared in 1 mM HCl containing 2 mM $CaCl_2$ (2 mg/mL). Transferrin is digested with chymotrypsin in a mass ratio of 1:60 (chymotrypsin: transferrin) and is allowed to proceed for 18 h at 30 ºC. The digestion reaction is terminated by snap freezing the sample at -20 ºC using liquid nitrogen.

MS Scanning Parameters. The Q-Exactive mass spectrometer (Thermo Fisher Scientific, San Jose, CA) uses LTQ Tune Plus software (version 2.7) with the following parameters: capillary temperature 350 ºC, positive mode MS, FT resolution 17,500, maximum injection 150 ms, automatic gain control target is 3 x 10^6, and S-lens RF 50. These settings resulted in a scan rate of approximately 3.8 scans/sec. Data are processed using Thermo Fisher Scientific Xcalibur (version 4.1) and Microsoft Excel (2025, Microsoft, Redmond, WA). Peaks were detected using a Gaussian 15-point auto smoothing and the genesis peak detection method. The MS ion transfer tube and the capillary outlet are grounded, creating a field-free region. The sweep gas cone is removed and the separation capillary and VSSI are installed on the Nanospray Flex™ interface (ES071, ThermoFisher Scientific) that is connected to the MS. The separation capillary is position is mounted on an additional micromanipulator (MT-XYZ, Newport Corp., Irvine, CA) to position the VSSI within approximately 2 to 2.5 mm of the MS inlet as measured using a magnifying camera (Dino-Lite Edge 3.0 digital microscope, Torrance, CA).

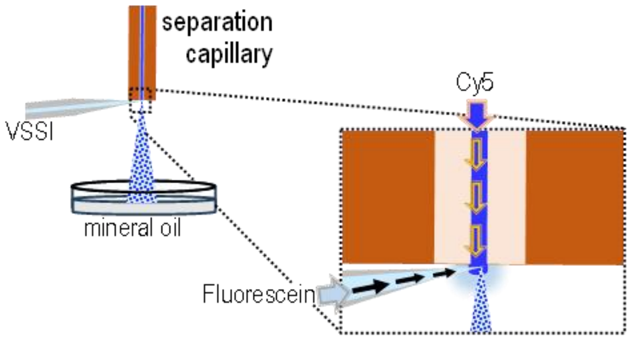

Figure S1. Conceptual depiction of the collection of VSSI drops with fluorescence microscopy. An aqueous solution of 20 μM Cy5 (λ_{ex} = 651 nm, λ_{em} = 670 nm) is pushed through the separation capillary at a flow rate of 31 nL/min, while 10 μM fluorescein (λ_{ex} = 498 nm, λ_{em} = 516 nm) is delivered through the VSSI probe at a flow rate of 650 nL/min. The VSSI drops formed after mixing at the interface are captured in mineral oil and imaged using a fluorescence microscope with red- and green-filters. The fluorescent images are converted to grayscale (see Fig S2A,B) and analyzed using ImageJ software. Both fluorescent dyes are present in each drop. A sample of 10 drops is shown in Figure S2C. The ratio of Cy5 to fluorescein, as evaluated using the grayscale intensity of each drop (Fig S2C and summarized in Table S1), is 0.9 ± 0.1, with larger drops having a higher ratio. This is attributed to the reduced contrast between the drop and the background as well as the increased edge artifacts for smaller drops.

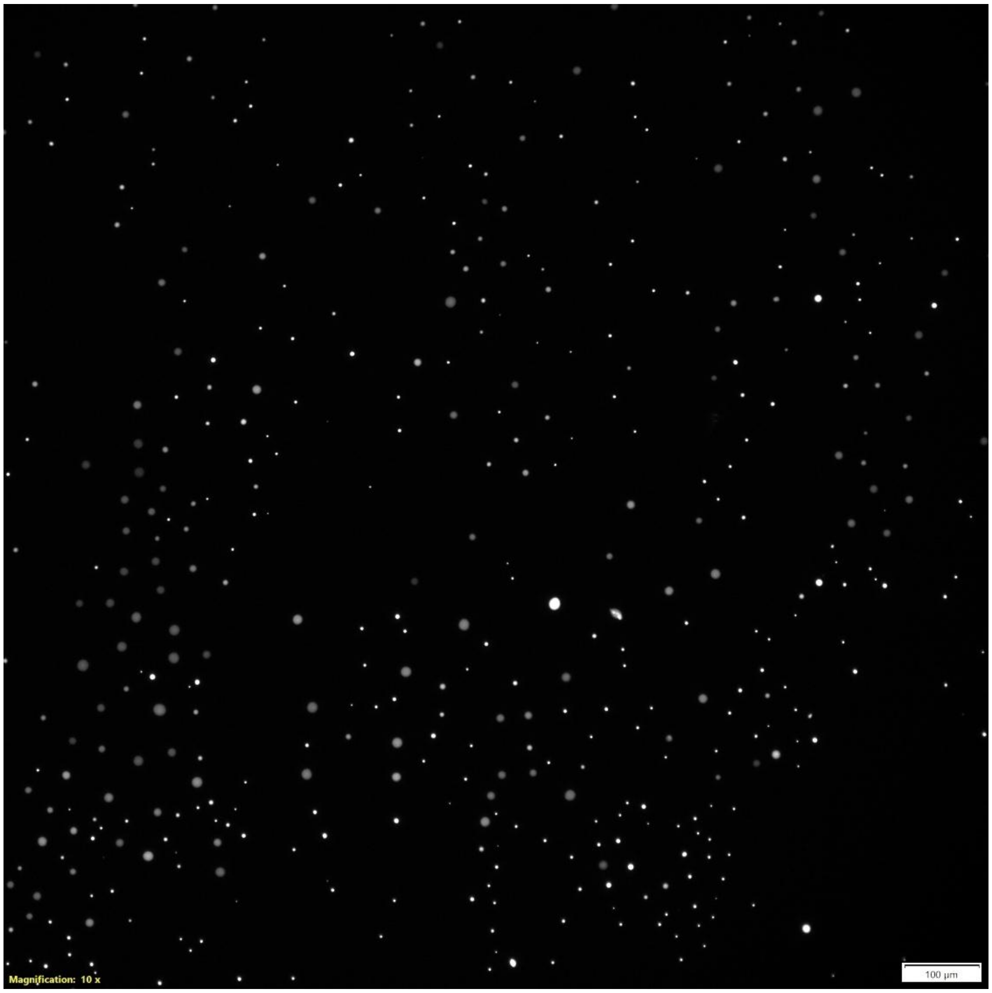

Figure S2A. Image of drops collected from capillary electrophoresis-VSSI using an Olympus IX 73 epifluorescence microscope with a green-fluorescence filter to detect fluorescein.

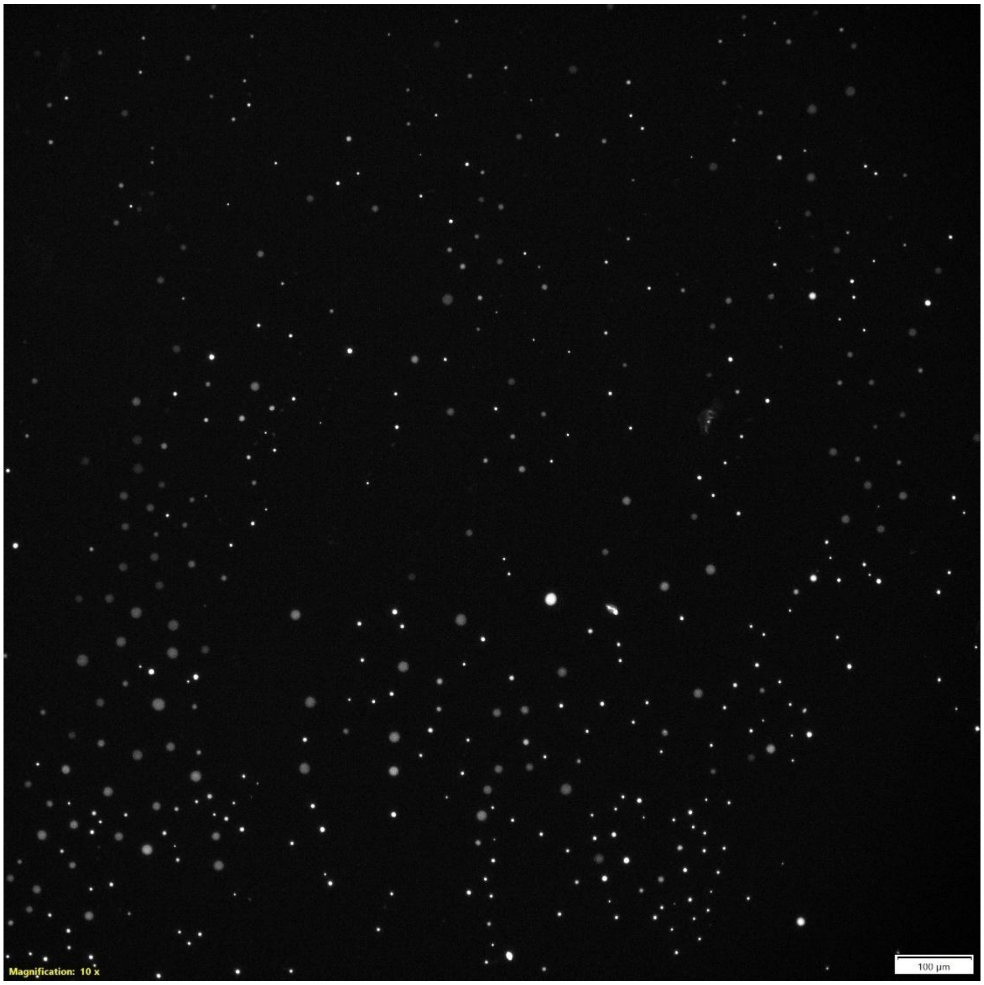

Figure S2B. Image of drops collected from capillary electrophoresis-VSSI using an Olympus IX 73 epifluorescence microscope with red-fluorescence filter to detect Cy5.

**Table S1:** Ratio of Cy5 to fluorescein grayscale intensity for data in Figure S2C							
**Drop #**	**Cy5 filter**			**Fluorescein filter**			**Ratio of mixing**
	area	mean	signal intensity x 10^3	area	mean	signal intensity x 10^3	
1	60.	141.433	8.5	34	172.676	5.9	0.69
2	52	153.654	8.0	34	175.353	6.0	0.75
3	124	95.395	11.8	156	80.506	12.6	1.06
4	44	167.045	7.3	52	150.538	7.8	1.1
5	36	155.444	5.6	34	139.412	4.7	0.85
6	60.	206.083	12	52	205.962	11	0.87
7	328	66.643	21.9	238	84.803	20.2	0.923
8	86	153.953	13	52	194.654	10.	0.76
9	44	171.182	7.5	37	153.189	5.7	0.75
10	60.	101.55	6.1	44	146.5	6.4	1.1

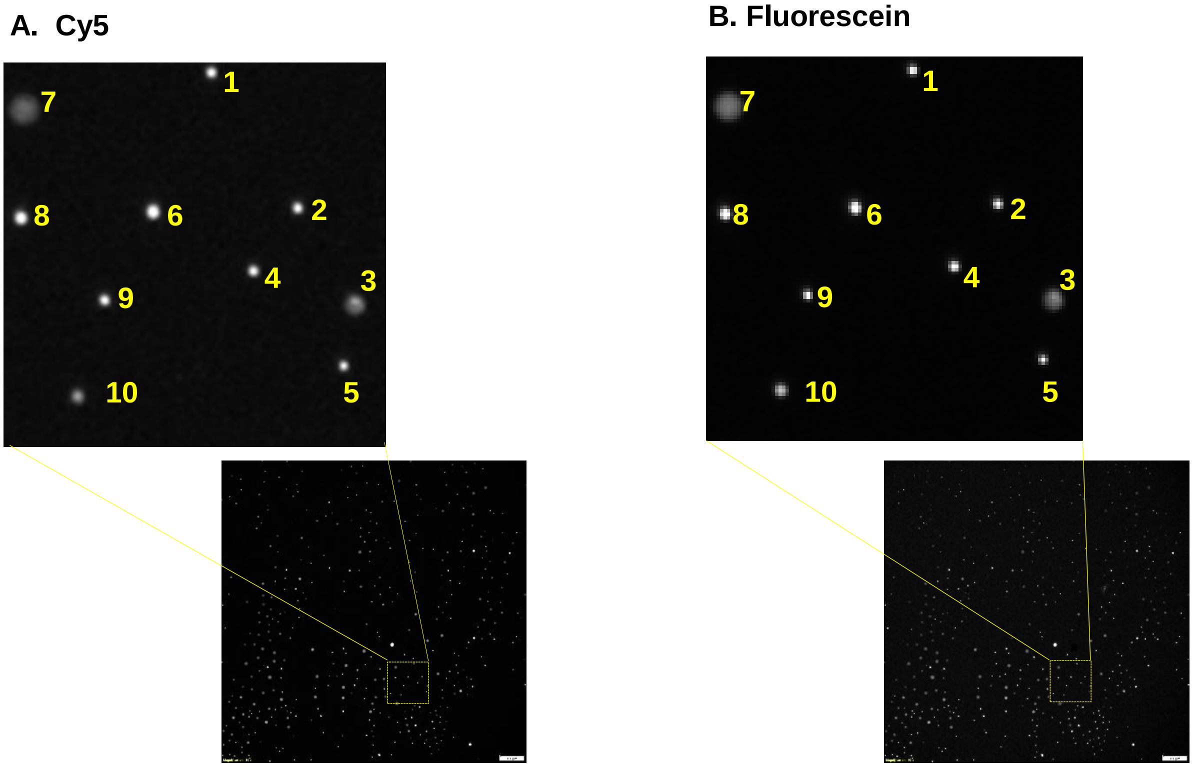

Figure S2C. Images of Cy5 and Fluorescein droplets are notated to associate drops in the images with drop number in Table S1

Run 45	Total Run Data points: 587		
Run 45 10 nM pin ace 20 kV 2s inj 10 kV run no stack.RAW			
Time	Noise	Time	Signal
1.170719	957.00914	1.322739	1161.401542
1.1750618	804.12554	1.327082	1430.756548
1.1794056	731.49862	1.331426	1974.648145
1.183749	766.11409	1.335769	2657.406106
1.1880923	881.4973	1.340113	3439.213054
1.1924354	965.34789	1.344455	4218.520868
1.1967787	1016.5889	1.348799	5097.019991
1.2011219	1066.1546	1.353142	6235.829149
1.2054648	1140.8521	1.357486	7699.589876
1.2098082	1243.591	1.361829	9338.067866
1.2141521	1367.7734	1.366172	10856.13752
1.2184947	1464.2285	1.370515	12049.02993
1.2228381	1496.6363	1.374858	12929.44849
1.2271814	1436.6233	1.379201	13500.14051
1.231525	1330.7463	1.383544	13899.38236
1.2358682	1248.6532	1.387888	13940.43837
1.2402153	1240.5732	1.392236	13614.79965
1.2445586	1241.0212	1.396579	12839.55655
1.2489028	1218.1433	1.400923	11709.93973
1.2532459	1163.0062	1.405266	10376.11875
1.2575895	1111.0289	1.409609	9004.82254
1.2619323	1125.2211	1.413952	7579.783194
1.2662756	1230.8027	1.418295	6116.601084
1.2706187	1371.3437	1.422638	4707.066264
1.2749624	1507.6808	1.426982	3498.612023
1.2793062	1613.3063	1.431325	2581.375203
1.283649	1686.0595	1.435668	2015.692295
1.2879923	1696.2291	1.440011	1712.753044
1.2923353	1628.5993	1.444354	1558.762205
1.2966792	1483.6124	1.448697	1382.585061
1.3010218	1315.8231	1.45304	1159.876357
1.3053652	1188.6811	1.457384	952.085271
Average	1242	Average	6601
SD	257		
LOD (nM)	1.44		
Data Points	32		

Run 46	Total Run Data points: 589		
Run 46 10 nM pin ace 20 kV 2s inj 10 kV run no stack.RAW			
Time	Noise	Time	Signal
0.901272	796.746663	1.3312646	1871.852289
0.9056153	1019.977291	1.3356074	2586.114915
0.9099584	1199.135919	1.3399537	3425.521983
0.9143011	1280.692198	1.3443095	4254.236762
0.9186444	1239.733157	1.3486525	4800.884474
0.9229878	1137.248301	1.3529953	5061.406156
0.9273306	1052.844641	1.3573387	5363.442618
0.9316736	989.410121	1.361682	6028.730495
0.9360168	910.016373	1.3660254	7171.405936
0.9403598	787.70551	1.3703688	8303.555562
0.9447031	704.521533	1.3747116	9032.620939
0.9490459	726.392203	1.3790546	9345.359538
0.9533897	847.649016	1.3833974	9606.082502
0.9577323	1002.405551	1.3877408	9973.496037
0.9620762	1146.515551	1.3920841	10289.14088
0.9664212	1236.48519	1.3964275	10270.08344
0.9707645	1216.393992	1.4007709	9720.029718
0.9751083	1077.071973	1.4051147	8755.881071
0.9794512	865.832881	1.4094592	7520.596631
0.9837944	710.368886	1.4138022	6000.276165
0.9881374	670.007125	1.4181459	4393.022139
0.9924813	714.281859	1.4224887	2912.735082
0.9968241	772.604373	1.4268321	1867.673196
1.0011674	798.698729	1.4311754	1414.503165
1.0055105	820.271923	1.4355188	1512.955167
1.0098543	853.560484	1.4398621	1840.618039
Average	945	Average	5897
SD	197		
LOD (nM)	1.19		
Data Points	26		

Run 47	Total Run Data points: 489		
Run 47 10 nM pin ace 20 kV 2s inj 10 kV run no stack.RAW			
Time	Noise	Time	Signal
1.1924162	1478.416053	1.322717	1050.371
1.1967594	1539.650529	1.32706	1192.343
1.2011031	1546.175205	1.331405	1387.146
1.2054457	1512.37946	1.335748	1565.19
1.2097886	1422.611289	1.340091	1827.55
1.2141319	1300.220189	1.344435	2231.931
1.2184753	1188.27413	1.348778	2922.685
1.2228183	1100.554467	1.353122	3906.527
1.2271621	996.309943	1.357465	5122.92
1.2315048	866.654005	1.361808	6315.899
1.2358478	718.790029	1.366151	7240.863
1.2401907	630.653673	1.370495	7636.034
1.2445336	637.71323	1.374838	7587.089
1.248877	722.590251	1.379181	7354.906
1.2532198	798.716563	1.383525	7475.777
1.2575674	852.418459	1.387868	7889.062
1.2619107	907.967409	1.392211	8283.922
1.2662538	950.178117	1.396555	8379.635
1.2705975	979.875115	1.400898	8065.758
1.2749403	1020.354738	1.405242	7389.622
1.2792836	1124.998408	1.409598	6378.132
1.283627	1306.742644	1.413941	5026.499
1.2879704	1482.929596	1.418284	3572.38
1.2923143	1578.842484	1.422627	2238.178
1.2966569	1576.235058	1.426971	1264.225
1.3010003	1456.843276	1.431314	642.5748
1.3053437	1263.503521	1.435657	454.8917
Average	1147	Average	4607
SD	317		
LOD (nM)	2.75		
Data Points	27		

Average LOD		SD	%RSD
1.8	±	0.8	47

Figure S3A. Direct VSSI data summary for limit of detection (LOD) calculation using signal and noise. The LOD was determined as LOD = ks (C/(H – h)), where k is the confidence factor (i.e., 3), s is the standard deviation of the noise, C is the sample concentration, H is the average signal of the analyte, and h is the average signal of the noise.

Run32c	Total Run Data points:421		
RUN 32c 20kV 2 s inj 0.01 uM bblockers in 50 mM AMAC pH 6.5 bGE !			
Time	Noise	Time	Signal
0.21433193	2029.7249	1.144472	2488.844971
0.22641113	3094.2368	1.156551	3259.54541
0.23849002	1003.444	1.168631	5126.022949
0.25056948	3640.8428	1.180712	9759.165039
0.26264867	694.76428	1.192792	10638.27832
0.27472842	3748.1807	1.204871	9829.15332
0.28680835	1544.9326	1.21695	5652.45752
0.29888767	2639.2983	1.229029	4630.912598
0.3109748	2137.7415	1.241108	3349.17627
0.32305383	1592.7	1.253188	2954.31958
0.33513275	2582.6191	1.265268	2513.590332
Average	2246	Average	5473
SD	959		
LOD (nM)	8.92		
Data Points	11		

Run 32	Total Run Data points: 429		
RUN 32 20kV 2 s inj 0.01 uM bblockers in 50 mM = AMAC pH 6.5 bGE =			
Time	Noise	Time	Signal
0.2020863	3362.35791	1.1443032	3348.334473
0.2141652	978.175232	1.1563824	11048.86035
0.2262447	3489.299316	1.1684616	11376.6709
0.2383235	1632.84436	1.1805407	11874.26953
0.2504069	2890.834229	1.1926199	8103.941406
0.2624869	1485.677612	1.2046991	5227.75293
0.2745661	3270.127686	1.2167784	4304.685059
0.2866459	2209.996582	1.2288577	5474.15625
0.2987257	3404.149658	1.2409369	3728.484863
0.310805	2331.065674	1.2530165	3700.24292
0.3228844	2461.333984	1.2650961	3038.779053
Average	2501	Average	6475
SD	826		
LOD (nM)	6.23		
Data Points	11		

RUN 32b	Total Run Data points: 421		
RUN 32b 20kV 2 s inj 0.01 uM bblockers in 50 mM AMAC pH 6.5			
Time	Noise	Time	Signal
0.32304927	1007.617981	1.144466	1701.293
0.33512913	1319.486816	1.156545	2327.127
0.34720851	1366.709106	1.168625	4502.583
0.35928812	1292.261475	1.180704	7040.195
0.37136687	1214.820557	1.192784	5908.958
0.38344635	1054.554932	1.204864	3660.023
0.39552607	1143.579956	1.216943	1967.987
0.4076059	1095.877686	1.229022	1948.696
0.41968472	1231.895874	1.241101	1939.466
0.43176427	1374.442627	1.253181	1853.534
0.44384315	1304.261475	1.26526	1852.991
0.45592852	975.799561	1.277345	2057.35
0.46800777	1358.949463	1.289424	2164.658
0.48008758	1647.820435	1.301504	1685.493
Average	1242	Average	2901
SD	179		
LOD (nM)	3.24		
Data Points	14		

Average LOD		SD	%RSD
6.1	±	2.8	46

Figure S3B. Previously published nanoflow sheath VSSI data summary for limit of detection (LOD) calculation using signal and noise. These data are taken from the original data associated with Table S4, CE-VSSI-MS 0.010 μM Pindolol in the supporting information associated with Elshamy, Y. S.; Strein, T. G.; Holland, L. A.; Li, C.; DeBastiani, A.; Valentine, S. J.; Li, P.; Lucas, J. A.; Shaffer, T. A. Nanoflow Sheath Voltage-Free Interfacing of Capillary Electrophoresis and Mass Spectrometry for the Detection of Small Molecules. Anal. Chem. 2022, 94 (32), 11329-1133. The LOD was determined as LOD = ks (C/(H – h)), where k is the confidence factor (i.e., 3), s is the standard deviation of the noise, C is the sample concentration, H is the average signal of the analyte, and h is the average signal of the noise.

Table S2. Migration times and peak areas of β-Blockers using CE-VSSI-MS

	**Pindolol**	**Oxprenolol**	**Atenolol**	**Timolol**	**Acebutolol**
**Migration time, $\bar{X}$ (RSD)**	7.68 (0.5)	7.84 (0.5)	7.90 (0.5)	8.04 (0.5)	8.43 (0.4)
**Peak Area, $\bar{X}$ (RSD)**	130000 (20)	180000(10)	60000 (20)	64000 (10)	110000 (10)

Measurements are based on *n=3* electrokinetically stacked injection of 10 nM of bata-blocker. Separations were obtained with the background electrolyte concentration at 50 mM ammonium acetate, and 1 mM ammonium acetate in the sample matrix. the separation capillary is 25 micrometers in internal diameter, total length, and effective length of 85 cm, with an applied voltage of 25 kV with a current of 5.9 μA, an injection voltage of 10 kV for 12 sec, with a VSSI auxiliary flow of deionized water.

Table S3. Migration times and peak areas of amino acids separated with suppressed electroosmotic flow using CE-VSSI-MS

	**Time (min)** $\bar{X}$ **(RSD)**	**Area** $\bar{X}$ **(RSD)**
Lysine (k)	8.8 (2)	200000 (5)
Arginine (R)	9.1 (2)	540000(8)
Histidine (H)	9.2 (3)	370000 (20)
Leucine (L)	12.1 (5)	600000 (60)
Asparagine (N)	12.3 (5)	77000 (6)
Threonine (T)	12.3 (5)	130000(10)
Glutamine (Q)	12.5 (5)	150000 (5)
Tryptophan (W)	12.6 (5)	230000 (9)
Glutamic acid (E)	12.7 (5)	120000 (6)
Phenylalanine (F)	12.8 (5)	700000 (10)
Proline (P)	12.8 (5)	600000 (20)
Tyrosine (Y)	13.0 (5)	150000 (30)

Measurements are based on *n=3* electrokinetically stacked injection of 0.5 µM (KRH) and 2.5 µM (LNTQWEFPY). Separations were obtained with the background electrolyte concentration at 2% formic acid, and 0.004 % formic acid in the sample matrix. the separation capillary is 25 micrometers in internal diameter, total length, and effective length of 85 cm, with an applied voltage of 25 kV with a current of 6.2 µA, an injection voltage of 10 kV for 6 sec, with a VSSI auxiliary flow of deionized water.

Table S4. Peak Areas obtained 50 μM MRFA, Angiotensin I, and Oxytocin and a background electrolyte of 2 % Formic Acid or 50 mM Ammonium Acetate (AmAc)

Peak areas of +1 of MRFA and sum of +1 and +2 of Ang I and Oxytocin (no Na adduct)

**2% formic acid pH 2.00**	**Average Peak Area, $\bar{X}$ (RSD)**		
	**MRFA**	**Angiotensin I**	**Oxytocin**
	1800000 (20)	1200000 (30)	1700000 (10)
**50 mM AmAc pH 6.78**	**Average Peak Area, $\bar{X}$ (RSD)**		
	**MRFA**	**Angiotensin I**	**Oxytocin**
	1900000 (6)	1000000 (20)	1300000 (20)

Peak areas of +1 of MRFA and sum of +1 and +2 of Angiotensin I and Oxytocin with Na adduct peak areas for corresponding charges

**2% formic acid pH 2.00**	**Average Peak Area, $\bar{X}$ (RSD)**		
	**MRFA**	**Angiotensin I**	**Oxytocin**
	2200000 (30)	1600000 (20)	3100000 (20)
**50 mM AmAc pH 6.78**	**Average Peak Area, $\bar{X}$ (RSD)**		
	**MRFA**	**Angiotensin I**	**Oxytocin**
	2500000 (5)	1300000 (20)	2200000 (20)

Sample is injected 5 psi 4s. The separation capillary is 25 micrometers internal diameter, total length, and effective length of 85 cm, with an applied voltage of 25 kV with a current of 5.8 μA and 6.6 μA with ammonium acetate and 2% formic acid, respectively. Auxiliary solution from the VSSI is deionized water. MS detection m/z range is 500 -1300. The peak areas for all peptides are comparable at pH 2.00 and pH 6.78 as determined by using Student's t-test (ρ =0.05) using +1 charge state of MRFA, sum of +1 and +2 charge state for both angiotensin I and oxytocin.

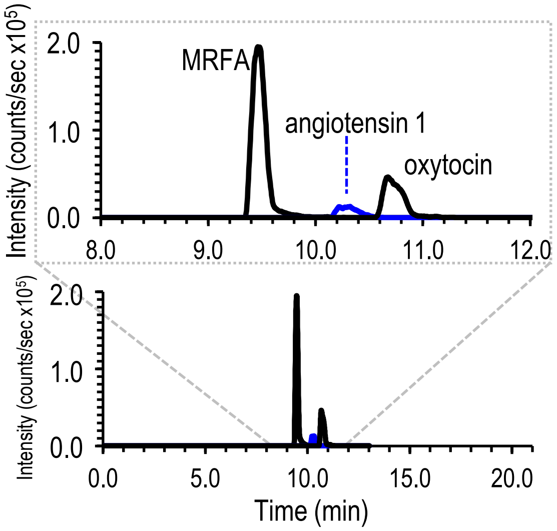

Fig S4. Peptide standard electropherograms obtained with a background electrolyte of 50 mM ammonium acetate at pH 6.78.

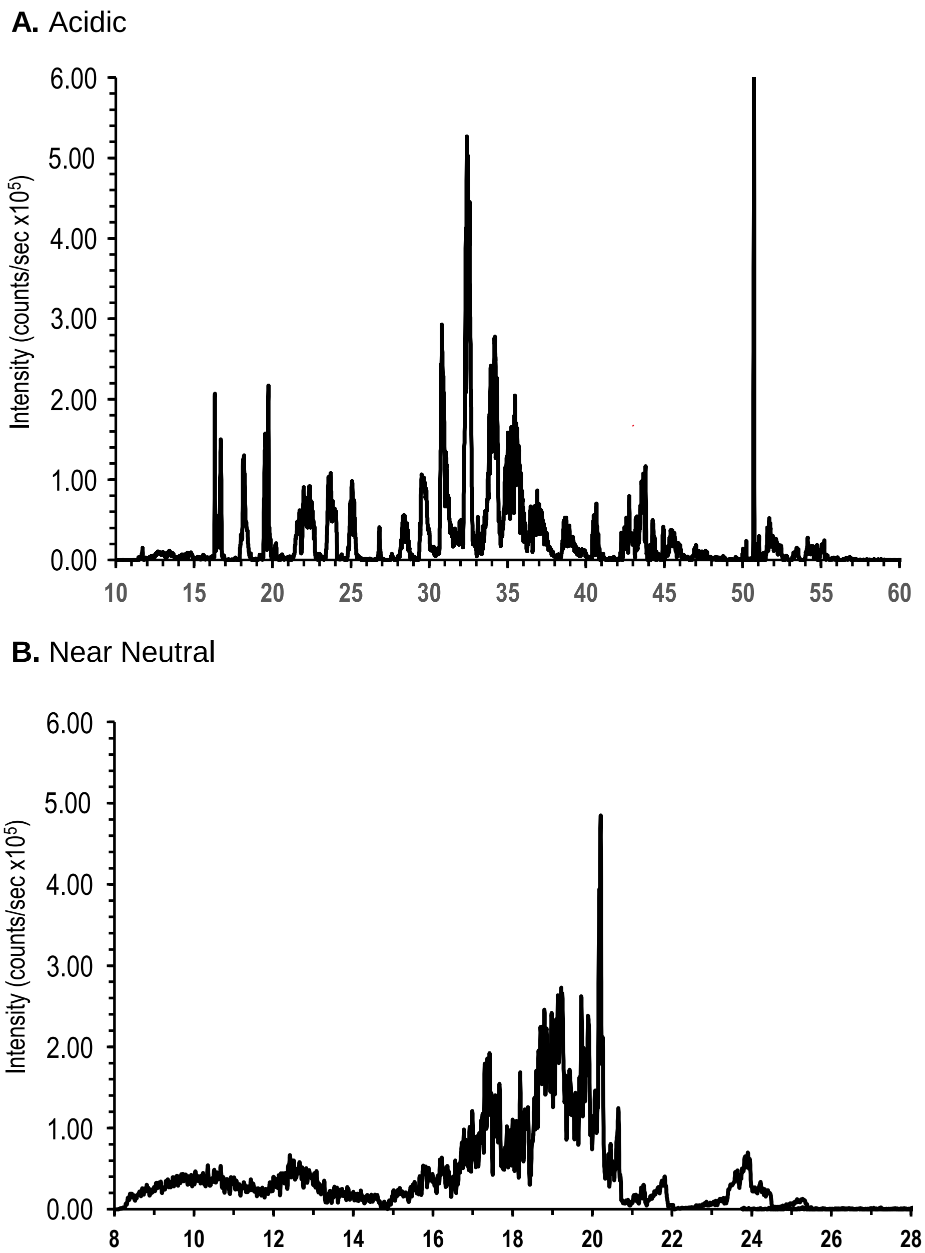

Figure S5. Electropherogram of peptides resulting from transferrin digestion with α-chymotrypsin using acidic pH (A) and near-neutral pH (B). The separation conditions are as follows: hydrodynamic Injection of 5 psi for 4 s, and the applied voltage is 25 kV. The BGE is 2% formic acid at pH 2 and 50 mM ammonium acetate at pH 6.8 for acidic and near-neutral pH, respectively. The VSSI fluid solution is 2.5 mM ammonium acetate for near-neutral pH and 0.1% formic acid for acidic pH, with a flow rate of 650 nL/min.

Table S5. Masses tabulated and observed for transferrin peptides using acidic and near-neutral background electrolyte				
**Expected mass $[M+H]^{+}$ [a]**	**Expected mass $[M+H]^{+}$ after reduction[b]**	**Peptide sequence[a]**	**Observed m/z with formic acid (acidic pH) $[M+H]^{+}$ [c,d]**	**Observed m/z with unadjusted ammonium acetate (near-neutral pH) $[M+H]^{+}$ [c,d]**
430.266		AVGAL	430.27	
504.285	561.3065	VCAVL	561.32	561.31
1071.5945		AVPDKTVRW	536.30	
1539.6566	1653.6995	CAVSEHEATKCQSF		
558.2453		RDHM	558.25	558.25
1936.0208	1993.0422	KSVIPSDGPSVACVKKASY		
1645.8213	1702.8428	DCIRAIAANEADAVTL		
375.1874		DAGL	375.19	375.19
368.1452		DAY	368.14	368.15
528.2776		APNNL	528.27	528.28
789.4505		KPVVAEF	789.45	789.45
1008.4632		GSKEDPQTF	1008.46	1008.47
1120.636		AVAVVKKDSGF		
374.2034		NQL	374.20	374.20
1086.5836	1143.6051	RGKKSCHTGL	1143.59	572.29*
633.3103		GRSAGW	633.31	633.31
626.3872		NIPIGL	626.39	626.39
1167.619	1224.6405	CDLPEPRKPL	612.32	
778.4093		EKAVANF	778.41	778.41
1568.6355	1682.6785	SGSCAPCADGTDFPQL		
363.1697	420.1911	CQL	420.19	420.19
840.3048	1011.3692	CPGCGCSTL		
381.1768		SGAF	381.18	381.18
363.206	420.2275	KCL	420.23	420.23
879.4206		KDGAGDVAF	879.42	879.42
831.4723		VKHSTIF	831.47	831.48
375.1874		ENL	375.19	375.19
1080.5068		ANKADRDQY	540.76*	
1236.5855		DNTRKPVDEY	1236.59	1236.59
615.2919	672.3133	KDCHL	672.31	672.32

1382.7209		AQVPSHTVVARSM		
618.3093		GGKEDL	618.31	618.31
318.1812		IW	318.18	318.18
873.3849		NQAQEHF	873.38	873.38
938.4941		GKDKSKEF	938.50	
840.421		SSPHGKDL	840.42	
761.3576		KDSAHGF	761.35	761.35
727.4283		KVPPRM	727.43	727.43
464.2173		DAKM	464.22	464.22
311.1237		EY	311.12	
786.4832		VTAIRNL		
2045.9419	2159.9848	REGTCPEAPTDECKPVKW		
306.1482	363.1696	CAL	363.17	363.17
778.3954		SHHERL	778.40	778.39
680.2708	737.2923	KCDEW	737.29	737.29
2527.2088	2641.2517	SVNSVGKIECVSAETTEDCI AKIM		
707.2665		NGEADAM	707.27	707.27
395.1561		DGGF	395.16	395.16
661.3701	718.3916	IAGKCGL	718.40	
427.2915		VPVL	427.29	427.29
496.2038		AENY	496.20	496.20
1542.6013	1599.6227	NKSDNCEDTPEAGY		
1201.715		AIAVVKKSASDL		
306.1448		TW	306.14	306.14
361.1718		DNL	361.17	361.17
1686.8856	1743.9071	KGKKSCHTAVGRTAGW		
474.2381		NIPM	474.24	474.24
1031.5203	1088.5418	NKINHCRF	544.78*****	
410.1558		DEF	410.16	
1365.6314	1422.6529	SEGCAPGSKKDSSL		
363.206	420.2275	CKL	420.23	420.23
253.0675	310.089	CM	310.09	310.09
333.1768		GSGL	333.18	333.18
1053.4306	1110.452	CEPNNKEGY	1110.45	1110.45
395.1925		TGAF	395.19	395.19
391.2122	448.2336	RCL	448.24	448.24
864.4461		VEKGDVAF	864.45	864.45
2003.0093		VKHQTVPQNTGGKNPDPW		
445.2769		AKNL	445.28	
668.2886		NEKDY	668.29	668.29
1193.5797		DGTRKPVEEY	1193.58	1193.58
557.25	614.2715	ANCHL	614.27	614.27

2456.3666	2513.3881	ARAPNHAVVTRKDKEACVHKIL		
809.4376		RQQQHL	809.43	405.22*
1100.4313	1157.4528	GSNVTDCSGNF		
848.4472		RSETKDL	848.44	
821.3822	878.4036	RDDTVCL	878.40	878.40
331.234		AKL	331.24	331.24
805.3587		HDRNTY	805.36	805.36
439.2187		EKY	439.22	439.22
497.1878		GEEY	497.19	
700.4352		VKAVGNL	700.44	700.43
881.4509	938.4724	RKCSTSSL	496.23*	
570.2228	627.2443	EACTF	627.24	627.24
428.2728		RRP	428.27	428.27

[a]Peptide sequences and masses obtained from simulation of cleavage using ExPASy PeptideMass Cutter, calculated as the monoisotopic masses of the occurring amino acid residues and giving peptide masses as $[M+H]^+$. The calculation is performed at https://web.expasy.org/peptide_mass/ as cited in Wilkins, M. R.; Lindskog, I.; Gasteiger, E.; Bairoch, A.; Sanchez, J.-C.; Hochstrasser, D. F.; Appel, R. D. Detailed Peptide Characterization Using Peptidemass – a World-Wide-Web-Accessible Tool. Electrophoresis 1997, 18 (3-4), 403-408.

[b]Peptide mass obtained from simulation of cleavage using ExPASy PeptideMass Cutter, calculated with cysteine treated with iodoacetamide as the monoisotopic masses of the occurring amino acid residues and giving peptide masses as $[M+H]^+$.

[c]Peptide mass is reported at $[M+H]^+$ unless otherwise noted

[d]Peptide mass is reported at $[M+2H]^{2+}$ when denoted with an asterisk (*)

Author contributions are written according to: Brand, A.; Allen, L.; Altman, M.; Hlava, M.; Scott, J., Beyond authorship: attribution, contribution, collaboration, and credit. Learned Publishing 2015, 28 (2), 151-155.

Author contributions are as follows:

YSE: Conceptualization, Methodology, Formal Analysis, Investigation, Data Curation, Writing- Original Draft preparation, Writing – Review and Editing, Visualization (Figures 2, 3, 4, and Supporting Information Figures and Tables).

LAH: Conceptualization (capillary grounding fitting, probe with auxiliary flow), Writing- Original Draft preparation, Writing – Review and Editing, Visualization (Figures 1, 2, 3, 4, 5, Supporting Information Figure S1C, S4), Supervision, Project Administration, Funding Acquisition

ELC: Validation, Investigation, Review and Editing.
